# The Effect of Concomitant Septoplasty and Turbinate Surgery on Nasality‐Related Voice Parameters

**DOI:** 10.1111/coa.14304

**Published:** 2025-03-18

**Authors:** Cevat Celenk, Burak Ulkumen, Onur Celik

**Affiliations:** ^1^ Otorhinolaryngology Clinic Manisa City Hospital Manisa Turkey; ^2^ Faculty of Medicine, Otorhinolaryngology Department Manisa Celal Bayar University Manisa Turkey

**Keywords:** acoustic, analysis, septoplasty, spectrography, turbinoplasty, voice

## Abstract

**Introduction:**

Our study aimed to reveal whether septoplasty and inferior turbinate reduction significantly impact the acoustic properties of nasalized syllables and alter subjective and objective voice parameters.

**Materials and Methods:**

Forty patients with nasal septal deviation and bilateral grade 2 ≤ inferior turbinate hypertrophy who underwent septoplasty and bilateral inferior turbinoplasty were enrolled. Participants completed the VHI‐10, VAS, and NOSE scales preoperatively and at 6 months postoperatively. Changes in VAS and NOSE scores were calculated as VAS^change^ and NOSE^change^ values. Voice recordings of the sustained vowel /a/ and the word /mini/ were analysed using MDVP. Acoustic analysis was performed with the sustained vowel /a/, and spectrographic analysis was conducted with the consonants /m/, /n/, and the vowel /i/ in /mini/. Recordings were taken preoperatively and at 6 months postoperatively. Statistical analysis compared pre‐ and postoperative values for significant changes using SPSS Version 21.0 (IBM Corp.; Armonk, NY, USA).

**Results:**

A statistically significant decrease in VAS and NOSE scores was observed at 6 months postoperatively (*p* < 0.05). No significant difference was found in VHI‐10 scores (*p* > 0.05). Acoustic analysis showed a significant change in pre‐ and postoperative F0 values (*p* < 0.05), but not in jitter, jitter%, shimmer, shimmer%, and NHR (*p* > 0.05). Spectrographic analysis revealed significant postoperative changes in the F3 and F4 formants of consonants /m/, /n/, and vowel /i/ in the word /mini/. A significant correlation was found between postoperative changes in F3 and F4 formant values for consonants /m/ and /n/ with the VAS^change^ value. For the NOSE^change^ value, a significant correlation was found only with the change in the F3 formant value for the consonant /m/.

**Conclusion:**

Nasal surgeries, particularly septo‐turbinoplasty, can influence voice timbre by modifying F3 and F4, which is of notable concern for professional voice users, such as singers and actors, due to the potential impact on the singer's formant cluster and overall vocal quality. Although it may not be appropriate to generalise for all rhinological surgeries, the significant changes in the F3 and F4 formants in a specific and refined patient group suggest that caution should be exercised in such surgeries, especially for professional voice users.

**Summary**

- The study investigates whether septoplasty combined with inferior turbinate surgery significantly alters voice parameters, particularly those related to nasality.
- Postoperative analysis revealed significant decreases in F3 and F4 formant frequencies for nasalized consonants (/m/, /n/) and the vowel /i/, indicating alterations in voice resonance.
- A significant increase in F0 was observed postoperatively for the vowel /a/, suggesting possible secondary effects of improved nasal airflow on vocal fold vibration.
- Improvements in nasal patency (measured by VAS and NOSE scores) correlated with changes in formant frequencies, emphasising the role of nasal airflow in voice production.
- The study highlights the potential impact of nasal surgery on vocal quality, particularly for professional voice users like singers and actors, who may experience subtle but meaningful changes in timbre and resonance.

## 1 Introduction

The human voice emerges from a delicate interplay among three sophisticated systems: the power source, vibratory mechanism, and resonators [1]. Expiratory air flow, which is produced by the lungs, constitutes the power source. Thereafter, this airflow causes the vibration of the laryngeal soft tissues, primarily the vocal folds. The transluminal pressure change, described by Bernoulli's principle, leads to the formation of the fundamental frequency (F0) [2, 3]. Ultimately, the phenomenon called resonance puts the last touches on the human voice. Resonance occurs in subregions of the upper airway like the pharynx, tongue, soft palate, nose, and paranasal sinuses. In essence, resonance relates to how the vocal tract influences the sounds as they travel through it, shaped by every component from the vocal folds to the lips and nostrils. The process of phonation generates a series of acoustic harmonics, which are then selectively amplified by the resonances within the vocal tract. This amplification can shape how the voice is perceived by both the listener and the speaker, affecting the overall perception of vocal quality. Therefore, it is expected that surgeries involving the mentioned anatomical regions will affect especially the resonance characteristics of the human voice. This assumption has been evaluated by some previous studies to a certain extent [4, 5, 6, 7, 8]. However, the effect of various surgeries of the upper airway on the formants (spectral peaks) of the human voice has not yet been fully elucidated [9].

Nasal obstruction is one of the most common complaints in otorhinolaryngology, primarily caused by nasal septal deviation and inferior turbinate hypertrophy, which often coexist [10, 11]. Thus, septoplasty typically includes inferior turbinate reduction to relieve obstruction [12]. The effectiveness of these surgeries is measured using subjective methods like the Visual Analogue Scale (VAS) and the Nasal Obstruction Symptom Evaluation (NOSE) scale, and objective methods such as rhinomanometry and acoustic rhinometry [13, 14, 15, 16]. The NOSE score, introduced by Stewart et al. in 2004, is highly reliable, sensitive, and specific for assessing nasal obstruction [14]. Our practice prefers the NOSE score for its simplicity and focus. The VAS also provides a subjective measure for evaluating symptoms and is widely used in medical research and other fields to assess self‐perception of nasal obstruction [15].

Human voice can be evaluated by both subjective and objective methods. While subjective methods like GRBAS and VHI‐10 are valuable for assessing overall voice status, they cannot precisely measure resonance characteristics [17]. Objective tests such as computerised voice analysis and spectrographic analysis provide detailed insights into resonance properties, evaluating parameters like F0, shimmer, jitter, and noise–harmonic ratio (NHR), and assessing voice formants [18]. Formants, resonant frequencies within the vocal tract, vary due to dynamic changes during vowel production and can shift after nasal surgery, especially in nasalized vowels [1, 19]. Thus, analysing the first four formants of nasalized vowels and nasal consonants helps assess the impact of nasal surgery on resonance [20, 21].

In this study, we aimed to test the hypothesis that a relatively standardised nasal surgery, specifically septoplasty combined with inferior turbinate surgery, would have a significant effect on the formant values of the nasalized vowels and consonants, as well as to ascertain whether there is a substantial change in both subjective and other objective voice parameters. There are numerous studies on the effect of nasal surgery on voice [11, 18, 22, 23, 24, 25, 26, 27, 28, 29, 30]. However, there are significant differences concerning the type of nasal surgery and the method of voice evaluation among these studies. Unlike the previous similar studies, we restricted the target population in terms of nasal patency, as described in the materials and methods section, to forestall possible bias.

## 2 Materials and Methods

### 2.1 Study Design and Sample

This study was designed as a prospective clinical trial and was conducted at the Voice and Speech Disorders Unit of the ENT Department, Manisa Celal Bayar University. It was approved by the Health Sciences Ethics Committee of Manisa Celal Bayar University Faculty of Medicine (protocol number: 27.03.2019/20.478.486). Written informed consent was obtained from every participant.

The study population consisted of patients who applied to the otorhinolaryngology outpatient clinic with the complaint of nasal obstruction between June 2019 and March 2020. Cases who underwent concomitant septoplasty and bilateral inferior turbinoplasty with the diagnosis of septal deviation and bilateral grade 2 and higher inferior turbinate hypertrophy were the target sample. Demographic data of all cases, including age, type of surgery, history of vocal abuse, and gastroesophageal reflux, was obtained. Exclusion criteria were determined as follows: (i) history of previous nasal surgery, (ii) nasal pathologies other than septal deviation and inferior turbinate hypertrophy (septal perforation, nasal polyposis, external nasal deformity, etc.) (iii) septal deviations requiring open technique (advanced caudal deviations, internal nasal valve issues, etc.) (iv) larynx and lung pathologies that may cause voice impairment, (v) history of surgery for the larynx, cleft lip and/or palate, (vi) cranio‐facial anomalies, (vii) speech disorders, and (viii) intellectual disability.

### 2.2 Preoperative Physical Examination

Detailed ENT examinations were done in all cases. Namely, nasal endoscopy, indirect laryngoscopy, and videolaryngostroboscopic examinations were performed. The nasal septum and inferior turbinates were evaluated by anterior rhinoscopy and nasal endoscopy before and after the application of topical decongestants. Grading of inferior turbinates was based on what percent of the total airway space was filled by them, as described by Camacho et al. [31]. Namely, we categorised them as Grade I (0%–25%), Grade II (26%–50%), Grade III (51%–75%), and Grade IV (76%–100%). We only included cases with bilateral grade II or higher inferior turbinate hypertrophy, based on this grading.

### 2.3 Surgical Technique

All patients underwent septoplasty and bilateral inferior turbinoplasty under general anaesthesia. Hemi‐transfixion incision was done after sub‐mucoperichondrial injection of %1 lidocaine and 1/100,000 adrenaline mixture. Next, mucoperichondrial flaps were created by a #15 blade and Freer elevator for total exposure of quadrangular cartilage, maxillary crest, and vomer. Septal deviation was corrected by using Blakesley forceps and chisel according to the location and type (bony/cartilaginous) of the deviation. Deviated bony or cartilaginous septal parts were removed after septotomy while taking care for preservation of the L‐strut. Excised cartilage was crushed and reimplanted for restoration of septal integrity.

**FIGURE 1:**
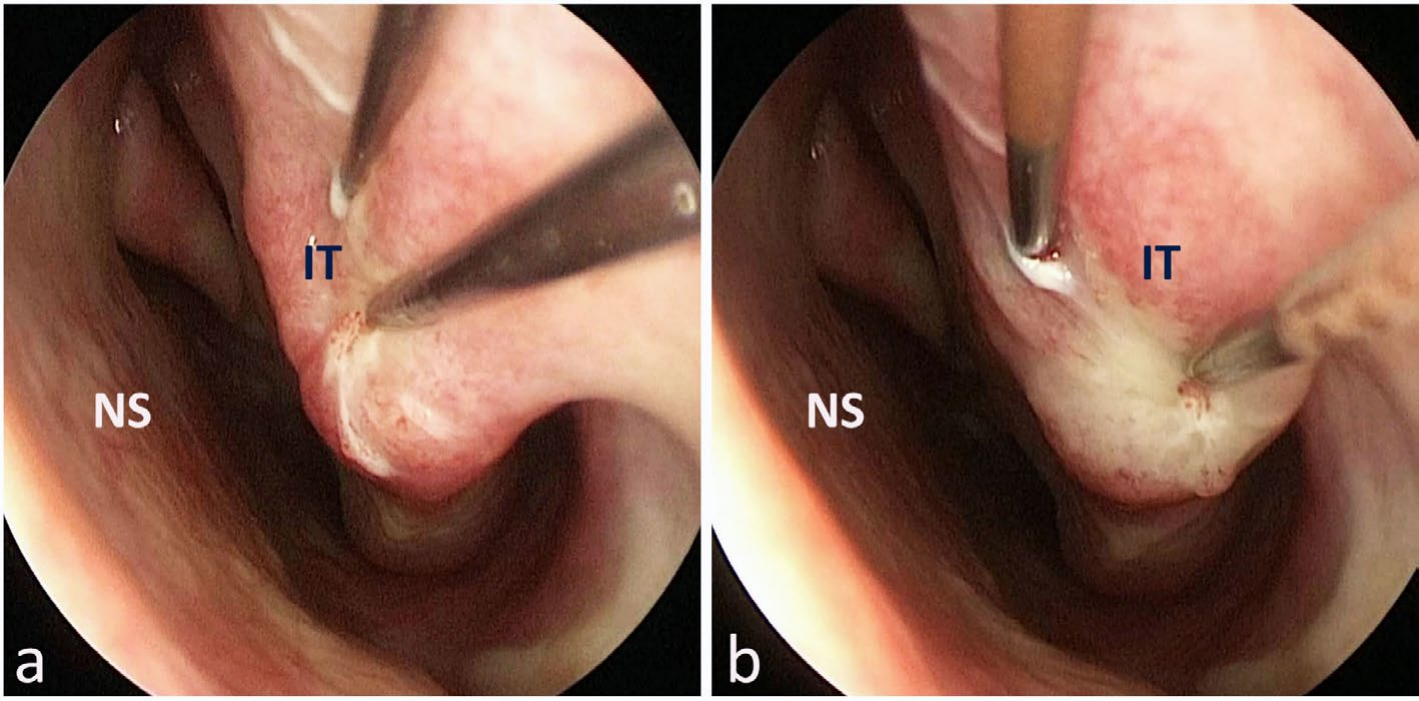
Peroperative endoscopic view of left nasal cavity (a) before and (b) after bipolar cautery application. Blanching of the turbinate mucosa can be seen (b). (NS, Nasal septum; IT, Inferior turbinate).

In every case, we performed out‐fracture and submucosal electrocauterization to both inferior turbinates. The out‐fracture was done by the blunt end of the Killian elevator. The inferior turbinate was first forced medially then laterally for a more consistent transposition. After that, both bipolar cautery tips were inserted into the anterior part of the inferior turbinate, with the tips roughly 3 mm apart (Figure 1a). The cautery was set at 25 watts. Cauterization was continued until blanching of the mucosa was observed (Figure 1b). The procedure was repeated for the posterior half of the inferior turbinate also. Intra‐nasal splints were inserted bilaterally and removed on the 5th day.

### 2.4 Evaluation of Voice and Nasal Patency

**FIGURE 2:**
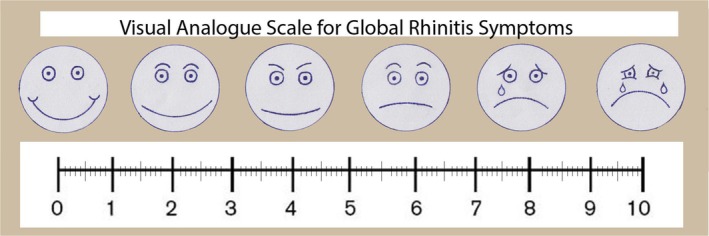
Visual analog scale for global rhinitis symptoms. The results of the VAS score were multiplied by 10 to establish a scale with a possible score of 100 for analysis.

All patients signed an informed consent form regarding the study and completed the nasal and voice‐related Patient Reported Outcome Measures (PROMs) before and after the surgery. The voice‐related PROM was the Turkish version of the VHI‐10 [32] questionnaire, while the nasal PROMs were the Turkish versions of the VAS (Figure 2) and NOSE (Table 1) questionnaires, administered just prior to the surgery and 6 months post‐surgery to evaluate nasal patency [33]. For the NOSE scale, patients circled the response closest to describing their current symptoms. The results of VAS and NOSE scores were summed and multiplied by 10 and 5, respectively, to establish a scale with a possible score of 100 for analysis. Subsequently, the changes between pre‐operative and post‐operative VAS and NOSE scores were calculated for each case, determining the VAS^change^ and NOSE^change^ values.

**TABLE 1:** The NOSE (Nasal Obstruction Symptom Evaluation) scale [26].

Over the past 1 month how much of a problem were the following conditions for you? Please circle the most correct response					
Symptoms^a^	Not a problem (A)	Very mild problem (B)	Moderate problem (C)	Fairly bad problem (D)	Severe problem (E)
1. Nose obstruction and stuffiness	0	1	2	3	4
2. Nose obstruction	0	1	2	3	4
3. Trouble breathing through my nose	0	1	2	3	4
4. Trouble sleeping	0	1	2	3	4
5. Unable to get enough air through my nose during exercise or exertion	0	1	2	3	4

^a^ Total score = A + B + C + D + E (Maximum score 20). Score out of 20 × 5 = Score out of 100.

Voice recording was performed in a quiet room with an ambient noise level of less than 30 dB. An Audio‐Technica AT2005USB Cardioid Dynamic USB/XLR Microphone (Audio‐Technica Inc., Ohio, USA) was used for the recordings. The distance between the microphone and the participant's mouth was 5 cm with an angle of 45°. The senior author demonstrated the task before the procedure. To avoid intra‐subject variability three separate recordings of sustained vowel /a/ were obtained at a habitual pitch, constant amplitude, and flat tone for at least 5 s. Additionally, given the fact that vowels adjacent to nasal consonants acquire nasality all patients asked to say the word /mini/ repeatedly for 5 s. This recording was used for spectrographic analyses. The sampling rate and resolution were 44.1 Hz and 16bit, respectively [32]. One second from the onset and offset of voice samples were cropped. Voice parameters like fundamental frequency (F0), absolute Jitter (Jitta), Jitter percent (Jitt), absolute Shimmer (ShdB), Shimmer percent (Shim), and noise to harmonic ratio (NHR) were determined upon the recordings of sustained vowel /a/ [34]. On the other hand, spectrographic analysis was done with the nasal consonants' /m/, /n/, and the nasalized vowel /i/, which are contained in the word /mini/. Whole voice recordings were repeated preoperatively and at the end of 6th month postoperatively. Next, we calculated the difference between each specific formant value for relevant vowels and consonants before and after the surgery for each case, determining the F^change^ values. Multi‐Dimensional Voice Program (MDVP) (Computerised Speech Lab (Kay Elemetrics Corporation, Lincoln Park, NJ, USA)) was used for acoustic analyses.

### 2.5 Statistical Analysis

Distribution of data were evaluated by the Shapiro–Wilk test. Variables with normal distribution were presented as mean (standard deviation [SD]), while non‐normal variables were reported as median (interquartile range [IQR]). The pre‐ and postoperative values of each parameter were compared by paired samples *t*‐test or Wilcoxon signed‐rank test according to the results of the Shapiro–Wilk test. The distribution of VHI‐10, NOSE, and VAS questionnaire values was not evaluated because they are categorised as an ordinal scale. For this reason, the Wilcoxon signed‐rank test was used in the evaluation of VHI‐10, NOSE, and VAS values. The correlation between the changes in VAS and NOSE values and the spectrographic voice parameters was analysed using Kendall's Tau test. Statistical significance was defined as *p* < 0.05. The Statistical Package for the Social Sciences (SPSS) Version 21.0 (IBM Corp.; Armonk, NY, USA) was used for statistical calculations.

## 3 Results

### 3.1 Descriptive Statistics

In this study, a total of 40 cases consisting of 6 females (mean age ± SD: 30.17 ± 7.86) and 34 males (mean age ± SD: 34.59 ± 11.05) who had undergone concurrent septoplasty and bilateral turbinoplasty were enrolled.

Only F4‐m^a,b^ and F2‐i^a,b^ of the data pairs showed normal distribution. For this reason, all the rest were analysed by the Wilcoxon signed‐rank test (Superscript “a” and “b” denote preoperative and postoperative, respectively). In this context, all the descriptive data were presented as median (interquartile range [IQR]) except for the abovementioned data sets.

### 3.2 Comparison of Nasal Patency

Comparison of pre‐ and postoperative NOSE and VAS scores revealed a statistically significant difference (Table 2).

**TABLE 2:** Comparison of pre‐ and postoperative nasal and voice Questionnaire values in terms of median (IQR) values of nasal and voice Questionnaires.

Questionnaire	Preoperative (Median [IQR])	Postoperative (Median [IQR])	*p*
VAS	60 (40–80)	10 (0–20)	< 0.001*
NOSE	65 (50–85)	12.5 (5–23.75)	< 0.001*
VHI‐10	2 (0.00–10.50)	1 (0.00–5.50)	0.059

* Statistically significant difference between measurements.

### 3.3 Comparison of Basic Voice Parameters

There was no statistically significant difference between pre‐ and postoperative VHI‐10 scores (Table 1). Besides, the comparison of pre‐ and postoperative acoustic parameters based on sustained vowel /a/ revealed no statistically significant difference except F0 (Table 3).

**TABLE 3:** Comparison of pre‐ and postoperative acoustic analyses upon the recordings of sustained vowel /a/.

/a/	Preoperative (Median [IQR])	Postoperative (Median [IQR])	*p*
F0 (Hz)	110.07 (101.34–127.168)	119.54 (105.85–140.26)	0.003*
Jitta	64.84 (41.09‐114.179)	68.64 (50.20–95.30)	0.485
Jitt	0.73 (0.44–1.59)	0.86 (0.58–1.36)	0.840
ShdB	0.28 (0.19–0.38)	0.27 (0.20–0.39)	0.839
Shim	2.98 (2.22–3.99)	3.09 (2.28–4.43)	0.933
NHR	0.14 (0.12–0.15)	0.13 (0.12–0.14)	0.273

* Statistically significant difference between measurements.

### 3.4 Comparison of Spectrographic Parameters Concerning the Word /Mini/

Comparison of formant values concerning the consonant /m/ revealed a statistically significant change in F3 and F4 (Table 4).

**TABLE 4:** Comparison of pre‐ and postoperative formant values of consonant /m/ in the word /mini/.

/m/	Preoperative (Hz)	Postoperative (Hz)	*p*
F1	3270.21 (3017.60–3574.90)^a^	3205.20 (3076.16–3454.08)^a^	0.946
F2	7397.09 (5800.74–7903.82)^a^	7413.39 (6022.98–8186.73)^a^	0.657
F3	10 726.66 (9626.49–11 253.73)^a^	9978.17 (9029.25‐10 667.01)^a^	0.016*
F4	13 305.39 ± 1383.60^b^	12 801.08 ± 1524.79^b^	0.031*

^a^ Median (interquartile range [IQR]).

^b^ Mean ± SD.

* Statistically significant difference between measurements.

Comparison of Formant Values Concerning the Consonant /n/ Revealed Statistically Significant Changes in F3 and F4 (Table 5)

**TABLE 5:** Comparison of pre‐ and postoperative formant values of consonant /n/ in the word /mini/.

/n/	Preoperative (Hz) (Median [IQR])^a^	Postoperative (Hz) (Median [IQR])^a^	*p*
F1	3255.89 (2976.55–3527.14)	3108.46 (2842.53–3677.05)	0.989
F2	7388.51 (60.92.95–7888.87)	6596.23 (5020.24–7762.48)	0.150
F3	10 346.55 (9839.49–11 111.96)	9707.02 (8384.44–10 542.35)	0.004*
F4	13.643.17 (12 578.14–14 584.89)	12757.92 (13 089.69 ± 1280.87)	0.007*

^a^ Median (interquartile range [IQR]).

* Statistically significant difference between measurements.

Comparison of Formant Values Concerning the Vowel /i/ Revealed Statistically Significant Changes in F3 and F4 (Table 6)

**TABLE 6:** Comparison of pre‐ and postoperative formant values of vowel /i/ in the word /mini/.

/i/	Preoperative (Hz)	Postoperative (Hz)	*p*
F1	3195.96 (2227.99–3794.67)^a^	2866.62 (273.79–4383.94)^a^	0.936
F2	7252.18 ± 1426.44^b^	7000.02 ± 2241.26^b^	0.483
F3	11 144.43 (9951.87–12 426.36)^a^	10 067.47 (8410.90–10 944.05)^a^	0.005*
F4	14 507.44 (13 493.76–15 608.25)^a^	13 825.08 (12 072.37–14 802.98)^a^	0.006*

^a^ Median (interquartile range [IQR]).

^b^ Medium ± SD.

* Statistically significant difference between measurements.

### 3.5 Evaluation of the Relationship Between the Nasal PROMs and the Change of Spectrographic Voice Parameters

The correlation between VAS^change^ and NOSE^change^ values and the changes in post‐operative specific formant values for the consonants /m/, /n/, and the vowel /i/ is summarised in Tables 7, 8, and 9, respectively.

**TABLE 7:** Correlation between the nasal PROMs and the change of formant values of consonant /m/ in the word /mini/.

/m/	VAS^change^ (*p* [*r*s]^a^)	NOSE^change^ (*p* [r]^a^)
F1^change^	0.707 (−0.043)	0.528 (−0.071)
F2^change^	0.725 (−0.040)	0.413 (−0.092)
F3^change^	0.010 (−0.295)*	0.033 (−0.240)*
F4^change^	0.009 (−0.298)*	0.175 (−0.153)

^a^ Correlation coefficient.

* Statistically significant correlation.

**TABLE 8:** Correlation between the nasal PROMs and the change of formant values of the consonant /n/ in the word /mini/.

/n/	VAS^change^ (*p* [*r*]^a^)	NOSE^change^ (*p* [*r*]^a^)
F1^change^	0.944 (−0.008)	0.761 (−0.034)
F2^change^	0.888 (−0.016)	0.413 (−0.092)
F3^change^	0.067 (−0.209)	0.361 (−0.103)
F4^change^	0.001 (−0.386)*	0.065 (−0.208)

^a^ Correlation coefficient.

* Statistically significant correlation.

**TABLE 9:** Correlation between the nasal PROMs and the change of formant values of the vowel /i/ in the word /mini/.

/i/	VAS^change^ (*p [r]* ^*a*^)	NOSE^change^ (*p* [r]^a^)
F1^change^	0.398 (0.097)	0.559 (0.066)
F2^change^	0.385 (0.099)	0.261 (0.127)
F3^change^	0.526 (−0.072)	0.963 (−0.005)
F4^change^	0.511 (−0.075)	0.426 (−0.090)

^a^ Correlation coefficient.

Considering the VAS^change^ value, a statistically significant correlation was found between the postoperative changes in F3 and F4 formant values during the vocalisation of the consonants /m/ and /n/ (Tables 7 and 8). Regarding the NOSE^change^ value, a statistically significant correlation was found only between the change in the F3 formant value during the vocalisation of the consonant /m/ (Table 7). On the other hand, during the pronunciation of the vowel /i/, no statistically significant correlation was found between either the VAS^change^ or NOSE^change^ values and the postoperative change in any formant value (Table 9).

## 4 Discussion

In the current study, we observed a significant decrease in the F3 and F4 parameters for the consonants' /m/, /n/, and the vowel /i/ (Tables 4, 5, 6), along with a significant increase in F0 for the vowel /a/. Spectrographic analysis of the word /mini/ showed significant decreases in F3 and F4 values post‐surgery. We also evaluated the relationship between the degree of nasal patency improvement and changes in spectrographic parameters. Significant changes in the F3 and F4 formants correlated with improvements in the VAS score, but not the NOSE score. Since both are PROMs, they primarily reflect patients' perception of patency. Therefore, the significant aspect is the noteworthy changes caused by our surgical intervention, particularly in the spectrographic parameters, rather than the correlation with nasal PROMs. The pronunciation of /mini/, especially the consonants /m/ and /n/, involves closed mouth resonance through sinonasal spaces. Surgical changes in these areas can affect nasalized syllable voice parameters, influencing voice perception. While F1 and F2 formant frequencies relate to vowel sound production, higher formants like F3 and F4 contribute to the unique characteristics and timbre of the voice [19]. The absence of changes in F1 and F2 suggests proper vowel sound pronunciation postoperatively. Changes in F3 and F4 suggest these parameters may influence the perception of nasality, though nasality perception is more linked to formant amplitude changes rather than frequency [1]. Not evaluating formant amplitudes is a study limitation. Therefore, in our study, we can suggest that the changes observed in F3 and F4 following rhinological surgery may have a more significant impact on professional voice artists when compared to the general population.

Changes in F3 and F4 can impact the singer's formant cluster, crucial for enhancing voice resonance and projection. The observed changes may have a more substantial impact on professional voice artists than the general population. As far as we know, the first spectrographic analysis related to rhinological surgery was conducted by Hong et al. [11] in 1997. They studied nasal polyposis cases without grading polyposis or nasal patency and found a decrease in F1 frequency and an increase in amplitude, but no change in other formants. Compared to septoplasty in our study, Hong et al.'s surgery likely caused a more dramatic change in nasal patency. Subsequent studies by Ozbal Koc et al., Goker et al., Gulec et al., Subramaniam et al., and Apaydın et al. [18, 27, 28, 29, 30] did not show significant changes in nasalized formants after rhinological surgery. These studies lacked objective nasal patency evaluations and/or homogeneity in the target population, making their results debatable. For example, Ozbal et al. [18] studied voice changes post‐septoplasty without specifying the surgical technique or condition of inferior turbinates. Goker et al. [27] compared submucosal turbinoplasty and radiofrequency treatment with a control group but did not assess nasal patency subjectively or objectively. Gulec et al. [28] and Apaydın et al. [30] used rhinomanometry to measure nasal obstruction but found no change in formant values. Subramaniam et al. [29] also did not evaluate nasal patency and found no spectrographic changes post‐septoplasty. Unlike these studies, our research included both septoplasty and turbinoplasty cases and ensured a homogeneous population by excluding deviations requiring open technique septoplasty and including only bilateral grade 2 or higher turbinate hypertrophy. We assessed nasal patency using both VAS and NOSE scores and found significant decreases in both, confirming a quantifiable relief in nasal obstruction (Table 2). While we did not use acoustic rhinometry or rhinomanometry, VAS and NOSE scores have been shown to be at least as effective in detecting nasal patency [15, 35].

The significance of the upper respiratory tract, including the nasal cavity and paranasal sinuses, in vocal resonance is widely acknowledged [7, 9, 23, 35, 36, 37, 38, 39]. A recent study by Vampola et al. using a novel method revealed that during nasalized vowel production, the acoustic energy of the first two formants decreases while that of the upper formants increases [1]. However, a reduction in acoustic energy is expected to decrease formant amplitude rather than frequency, which was not evaluated in our study. Rhinological surgery is anticipated to impact vocal resonance, particularly during nasalized syllable production. Assessing this impact is complex due to the diverse range of rhinological procedures and techniques. For example, inferior turbinate surgery and septoplasty vary in methods and equipment used [27, 40, 41]. This variability affects nasal cavity topography and cross‐sectional area. Additionally, factors such as intranasal mucosa condition, velopharyngeal closure status, and linguistic variations complicate assessments [9]. Consequently, subjective and objective voice evaluations alone may inadequately capture nasality‐related changes due to nasal surgery. Therefore, spectrographic analysis of formants is crucial to objectively determine the impact of nasal surgery on nasality‐related vocal resonance, especially during nasalized vowel production [18, 42].

Several studies have evaluated the impact of nasal surgery on nasal resonance using nasometry instead of spectrographic analyses [22, 23, 24, 25, 26]. For example, Kim et al. assessed the effect of septoturbinoplasty and bilateral endoscopic sinus surgery on acoustic voice parameters using MDVP and nasalance with a nasometer [22]. They found an increase in nasalance in the early postoperative period, which returned to preoperative values, attributing this to temporary postoperative sinonasal mucosal crusting. However, it's unclear why long‐term nasalance didn't remain elevated despite expected changes in nasal airflow and patency. Yang et al. reported a significant increase in nasality following unilateral endoscopic sinus surgery [24], highlighting a lack of consensus on the effect of such surgeries on nasal resonance. Kim et al. and Kasemsiri et al. also found increases in nasality after transsphenoidal surgery [23, 25], but this procedure alters intranasal morphology differently from common nasal surgeries like septoplasty and turbinoplasty, making generalisations difficult. Bakhshaee et al. used a nasometer to study nasalance changes after rhinoplasty but found no change, likely because rhinoplasty is primarily for cosmetic rather than obstructive reasons [26].

While our study did not uncover any notable modifications in fundamental acoustic parameters other than spectrographic analysis, we did observe a statistically noteworthy elevation solely in the F0 parameter. It is worth noting that only a handful of previous studies have reported a significant alteration in F0 following different types of nasal surgeries [7, 23, 38]. Kim et al. found a significant decrease in the F0 value after endoscopic endonasal transsphenoidal surgery [23]. In contrast, Mora et al. and Atan et al. reported a significant increase in F0 after septoplasty, like the results of the current study [7, 38]. The F0 value, being a direct result of vocal fold vibration, is unlikely to be impacted by nasal surgery. Any reported changes in F0 may be attributed to the indirect influence of nasal obstruction on vocal fold vibration. Namely, insufficient airflow through the nose can lead to disruption of lower airway humidification and thermoregulation. Additionally, the quantity and quality of particles that pass into the lower airway also change [39]. All these factors can affect the vocal fold mucosa in the short and long term, potentially influencing the F0 and harmonics. We believe that the increase observed in the F0 value in our study is related to these secondary effects.

In summary, we did not observe any significant changes in basic acoustic parameters such as Jitta, Jitt, ShdB, Shim, and NHR, except for F0. On the other hand, we detected a significant decrease in F3 and F4, which are the spectrographic parameters that may correlate with the timbre of voice during nasalized syllables. We believe that our results are more reliable compared to other similar studies, as we have been meticulous in considering the change in nasal obstruction in our target population. On the other hand, as we did not compare the amplitude values of the formants, we may have failed to detect possible postoperative changes in F1 and F2. Therefore, it would be appropriate for future studies to compare not only the frequencies of the formants but also their amplitude values.

## 5 Conclusions

In this study, we explored the impact of rhinologic surgery on vocal parameters, revealing significant alterations in the formant frequencies F3 and F4 for certain nasalised syllables and an increase in the fundamental frequency F0 for the vowel /a/, along with a direct correlation between improvements in VAS scores and formant values. These findings suggest that nasal surgeries, particularly septo‐turbinoplasty, can influence voice timbre by modifying F3 and F4, which is of notable concern for professional voice users, such as singers and actors, due to the potential impact on the singer's formant cluster and overall vocal quality. Although it may not be appropriate to generalise for all rhinological surgeries, the significant changes in the F3 and F4 formants in a specific and refined patient group suggest that caution should be exercised in such surgeries, especially for professional voice users. Therefore, it is essential to provide accurate information to professional voice users regarding the potential impact of these surgeries on their voice. Surgeons should assess the patient's nasal obstruction and inferior turbinate size and collaborate with speech therapists to manage any post‐operative voice changes effectively. Additionally, the study highlights the importance of using both subjective and objective measures to assess nasal patency and its correlation with vocal changes post‐surgery. Future research should aim to broaden the scope of investigation to include more diverse surgical techniques and patient populations, enhancing our understanding of the complex relationship between nasal anatomy and voice. This study also underscores the critical need for interdisciplinary collaboration in the fields of otolaryngology and voice training to better assess and address the implications of nasal surgeries on vocal performance.

## Author Contributions

Cevat Celenk was involved in hypothesis development, surgical procedures, voice analysis, statistical analysis, and manuscript writing. Burak Ulkumen was involved in the study design, hypothesis development, surgical procedures, voice analysis, statistical analysis, manuscript writing, and finalization. Onur Celik was involved in the study design, hypothesis development, surgical procedures, statistical analysis, manuscript writing, and finalization.

## Ethics Statement

It was approved by the Manisa Celal Bayar University Faculty of Medicine Health Sciences Ethics Committee (protocol number: 27.03.2019/20.478.486). The wet‐signed ethics approval form has been uploaded to the system as Supporting Information.

## Consent

Written informed consent was obtained from every participant. A sample of the written patient consent form has been approved by the relevant ethics committee and can be obtained from the ethics committee upon request.

## Conflicts of Interest

The authors declare no conflicts of interest.

### Peer Review

The peer review history for this article is available at https://www.webofscience.com/api/gateway/wos/peer‐review/10.1111/coa.14304.

## Supporting information


**Data S1.** Supporting Information.

## Data Availability

Statistical data related to the patients have been uploaded as a Supporting Information in SPSS format to the journal's online platform during the manuscript submission process. Surgical, imaging, and voice recordings of the patients included in the study are stored in our clinic's archive. These can be provided by the corresponding author upon request.
